# Introduction of digital speech recognition in a specialised outpatient department: a case study

**DOI:** 10.1186/s12911-016-0374-4

**Published:** 2016-10-18

**Authors:** Christoph Ahlgrim, Oliver Maenner, Manfred W. Baumstark

**Affiliations:** 1 Center for Medicine, Institute for Exercise- and Occupational Medicine, Medical Center – University of Freiburg, Hugstetter Str. 55, 79106 Freiburg, Germany; 2 Klinikrechenzentrum, Abteilung Systemtechnik, Medical Center – University of Freiburg, Hugstetter Str. 55, 79106 Freiburg, Germany; 3 Faculty of Medicine, University of Freiburg, Freiburg im Breisgau, Germany

**Keywords:** Operations, Digital dictation, Speech recognition, Documentation as a topic, MSC 62-07, MSC 68N99

## Abstract

**Background:**

Speech recognition software might increase productivity in clinical documentation. However, low user satisfaction with speech recognition software has been observed. In this case study, an approach for implementing a speech recognition software package at a university-based outpatient department is presented.

**Methods:**

Methods to create a specific dictionary for the context “sports medicine” and a shared vocabulary learning function are demonstrated. The approach is evaluated for user satisfaction (using a questionnaire before and 10 weeks after software implementation) and its impact on the time until the final medical document was saved into the system.

**Results:**

As a result of implementing speech recognition software, the user satisfaction was not remarkably impaired. The median time until the final medical document was saved was reduced from 8 to 4 days.

**Conclusion:**

In summary, this case study illustrates how speech recognition can be implemented successfully when the user experience is emphasised.

**Electronic supplementary material:**

The online version of this article (doi:10.1186/s12911-016-0374-4) contains supplementary material, which is available to authorized users.

## Background

The introduction of a speech recognition solution for medical documentation in the healthcare sector can improve productivity, as expressed by letter-turnaround-time [1], without remarkably impairing the quality of patient records [2]. It has, however, been described that introducing speech recognition software might impair user satisfaction leading to demotivation of the physicians using the software [3] and also might impose more workload on physicians [4]. As a consequence of a lack of motivation and hence, less involvement with the software, low contribution rates of speech recognition for the final text might occur [3], which could partially explain the heterogeneity of observed productivity gains when speech recognition is used [2].

In this article, we aim to provide a case study on the steps to successfully implement speech recognition software in a highly specialised university outpatient department focusing on the prearrangements that appear to be beneficial concerning productivity of the software and user motivation as the importance of this “predesign stage” has been emphasised before [5].

### Preliminary considerations

As indicated by Alapetite et al. [3], a change initiative concerning speech recognition deals with implementation of novel technology for which user satisfaction and objective functionality are strongly linked. This means that users who do not get involved with such software might not acquire the necessary skills to fully benefit from a speech recognition solution. Hence, user experience was brought into central focus during the preliminary considerations when planning implementation in our department. In Kotter’s change management framework [6], it is argued that implementation of change is much facilitated by creating “short-term wins” for users. In our context, “short-term wins” are understood as the immediate experience of a versatile, adaptive software, which can be used productively (from the user perspective) after a relatively short period of time. When implementing a novel technology maximisation of the initial performance has generally been identified as beneficial [7]. In our context, this translates into a high a priori speech recognition rate, so that physicians will perceive the software to be productive. A characteristic of our department is the very specific purpose of the department with a focus on elite sports and a close link to sports sciences. For a high a priori speech recognition rate, creation of an adapted dictionary for our specific purpose in addition to the general dictionary of the discipline (internal medicine) appeared necessary to account for the specific vocabulary used for documentation.

A vocabulary learning function of the speech recognition software is mandatory for a clinical speech recognition system [8]. From an organisational perspective, transfer of acquired knowledge between employees in the sense of shared learning [9] appears advantageous. One of our initial goals was to establish an environment for knowledge transfer concerning the novel vocabulary that was acquired by use of speech recognition during the initial adaptation phase. We aimed at making sure that all new words one user had taught the software in the implementation phase were available for all other users (meaning shared knowledge acquisition), also in order to feel that the software is improving. We expected that this would also help in creating social cohesion of the users during the introduction phase of the software. The version of the speech recognition package (SpeaKING ver. 7.1x) that was used did not allow easy central vocabulary management when used without central typists.

Based on this analysis, we considered a) providing a high initial speech recognition rate due to a specific dictionary for our department and b) an appreciable, rapid vocabulary learning function as “short-term wins” for users.

Information in this article can be classified using the usability framework for speech recognition technologies that was proposed by Dawson et al. [10], which relates usability design context to utilisation context. By aiming to improve the “technology characteristics” of the software in a favourable way, we hoped to meet user expectations (as part of the usability design context). Other aspects of the framework (user characteristics, task characteristics, technology characteristics and usability design context) are provided where applicable. Concerning the utilisation context, more focus is placed on user satisfaction but the concept of efficient utilisation will also be used.

## Methods

### Speech recognition software

The speech recognition solution used in our department was SpeaKING (ver. 7.1x, Mediainterface, Dresden, Germany). The decision for this software package was made by the board of directors of our university medical center. Technical details of the software like feature vectors and sampling frequency are not known to the authors.

Advantages and disadvantages of the software solution will not be discussed in this case presentation. In brief, in our context the software package has the technology characteristics of a client-server-based speech recognition application which provides direct transcription of the dictation at the cursor position. Specific speech recognition performance data, as discussed below, is currently not made available to the end user by Mediainterface.

### Our sports medicine department and the need for a specific dictionary

The user/task characteristics of the physicians involved in this study are those of experienced internal medicine residents and physicians who will use the software to write their discharge notes. All physicians working at the department and using the speech recognition software (*n* = 8) could participate in this study on a voluntary basis. Each doctor had a personal desktop computer with a local client of the speech recognition software installed. In our department, the language of the doctor’s notes is German. For our department, the inbuilt German dictionary for internal medicine of SpeaKING is used (due to the clinical background of our department).

When integrating the dictation process into a process map, no sub tasks are altered concerning the authorisation of a first draft for the document. Previously, doctors were able to use a typist office for their dictations. Due to closure of this service in June 2015, the physicians could now use speech recognition for their documentation. This means that the text writing process was shifted from typewriters to the physicians. With speech recognition, they prepare the complete, partially structured document for a patient which is then printed and handed over for review by at least one other physician. The documentation is usually prepared after the outpatient has left the department and is then sent by conventional mail to the referring physician and has a length of 1–2 written pages (11 pt.). Due to the specific context of the department, the vocabulary of the letters contains expressions from internal medicine and exercise physiology.

In order to provide a more customised dictionary covering the specific vocabulary, our aim was to analyse all letters written after 2009 (*n* = 13,430) to extract the relevant vocabulary. This has been proposed as the “pragmatic way” for establishing German medical thesauruses in clinical speech recognition [11]. Our approach is described briefly in the following: First, all letters, present as.doc and.rtf files, were converted to text files using command line LibreOffice (Version 5.0x, Mozilla Public License v2.0) [12]. Words were extracted from the content part of the letters using standard Linux tools in order to create a list of all words used in these letters. Words after punctuation marks (:.;) were omitted to avoid incorrect capitalisation. Further processing of this word list was preformed using R (Version 3.2.2, R Foundation for Statistical Computing, GNU General Public license) [13]. In total, 62,097 words were obtained. All words that had been used less than five times and words starting with digits were excluded from the word list. The resulting 15,883 words were then matched against the word list of the inbuilt internal medicine vocabulary of the speech recognition software, resulting in 2,060 words. This means that the inbuilt dictionary covered 87 % of the vocabulary used in our letters. To automatically check for typing errors, an analysis based on “Hunspell” [14] and the statistics package R was used based on Hornik and Murdoch’s considerations [15]. The resulting wordlists (*n* = 1,273) were revised manually. This resulted in a final list of 635 words, which were then included in the all-user vocabulary before the software was implemented at the department to increase the a priori speech recognition rate. For details of the technical procedure, see the supplemented material [see Additional file 1].

### Vocabulary learning function

As indicated above, the capability of a shared learning function was identified as necessary before implementation of the software in order to allow “knowledge transfer” between individual users to enable a teamwork experience. The inbuilt capacities of the software were therefore enhanced by the IT department of our hospital through automated standard-query-language (SQL) scripts in order to allow for automated extractions of single user vocabulary to enable regular updates of the general word list to be conducted based on the single-user vocabularies. These updates of the general vocabulary were conducted throughout the first 4 weeks after implementation of the software at a high frequency. After this period the general updates of the vocabulary were discontinued because we assumed that lexical originality [16] of the individual doctors’ notes was then outbalancing the benefits for all users.

### Additional concerns

A step-by-step manual on how the software (with our custom-made vocabulary learning functionality) is to be used was prepared and presented during a launch meeting in the first week of July 2015. In the 4 weeks after the software launch, close individual monitoring of the users was granted to allow quick troubleshooting as this had been identified as important by others [8].

### Evaluating success

User expectations and satisfaction [10] were assessed using a German translation of a quantitative descriptive questionnaire for user satisfaction with speech recognition [3] (Questions: 1, 5, 6, 8, 9, 10, 11, 12, 13, 14). The questionnaire has been evaluated in a recent review as an appropriate measurement for user expectation and experience [2] and thus appeared justified for our purposes. Questions 1, 5 and 6 evaluate the general attitude towards the introduction of speech recognition software. Questions 8, 9, 10, 11 and 12 evaluate the impact of speech recognition on the process of medical record keeping from the perspective of a physician. Questions 13 and 14 evaluate the direct impact of the introduction of speech recognition software on the physician. Assessing these last two items appears to be of special importance because a recent review critically examining the risks and benefits of speech recognition for clinical documentation emphasises that overall improvements after introduction of speech recognition solutions may mask additional document creation effort imposed to the clinician [4], which might impact on other clinical tasks and even error rates [17]. The questionnaire was handed to all users before the implementation of the software and after 10 weeks of software use. Users returned the questionnaire anonymously. The results are presented graphically due to the low number of physicians at the department using the software (*n* = 8).

Impact of availability of the dictionary for a priori speech recognition capability was studied *post hoc* by analysing 1,279 discharge notes that were created when the new dictation software package was available from August 2015 to March 2016. Using command line Linux commands, the number of occurrences of dictionary words in each document was analysed. The number of occurrences equals the number of corrections one user would have initially made in case no specific dictionary had been available when the software was introduced.

The time of creating the final draft of the medical document was assessed using the parameter “time to letter finalised” which was the period between the time the patient presented himself to the outpatient department until the time that the respective letter was finalised. The analysis of this lag was conducted by analysing the discharge letter files in R. The date of presentation was extracted from the letter using R and bash scripts. Letters were converted to text as described above. The date when the letter was finalised was the ‘last modified’ file date of the document. This analysis was conducted for all available letters. A period from 2009 to 2011, which was the period where “time to letter finalised” was lowest at our department when human typists were part of the process, was chosen as a benchmark.

## Results

### Dictionary enhancement

**Fig. 1:**
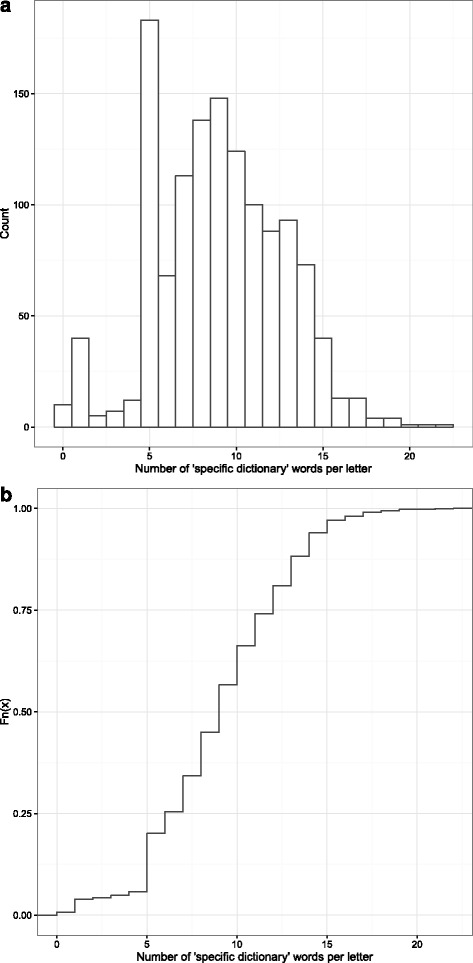
A + B. Histogram (**a**) and cumulative distribution function (**b**) of occurrences of words from the specific dictionary in 1,279 discharge notes created between August 2015 and March 2016. The X-axis gives the number of occurrences of dictionary words in letters from the observation period. In A, the Y-axis provides the number of documents. In B the Y-axis gives cumulative probabilities, the Figure can be interpreted as follows: the chance of dictating a letter that does not contain a word (x = 0) from the specific dictionary is less than 1 %

A list of 635 individual words was obtained from analysing the available documentation (all patient discharge notes from the last 6.5 years). These terms were included in the all-user vocabulary before the software was implemented. The 4-week learning period provided about 300 further words to the dictionary of the department. The *post hoc* analysis of dictionary words occurring in discharge documents that were created with availability of speech recognition is illustrated in Fig. 1 A + B. The cumulative distribution of occurrences reveals that practically all documents from the observed period contained dictionary words. On average, nine to ten words from the dictionary appear in current letters from our department.

### User satisfaction

**Fig. 2:**
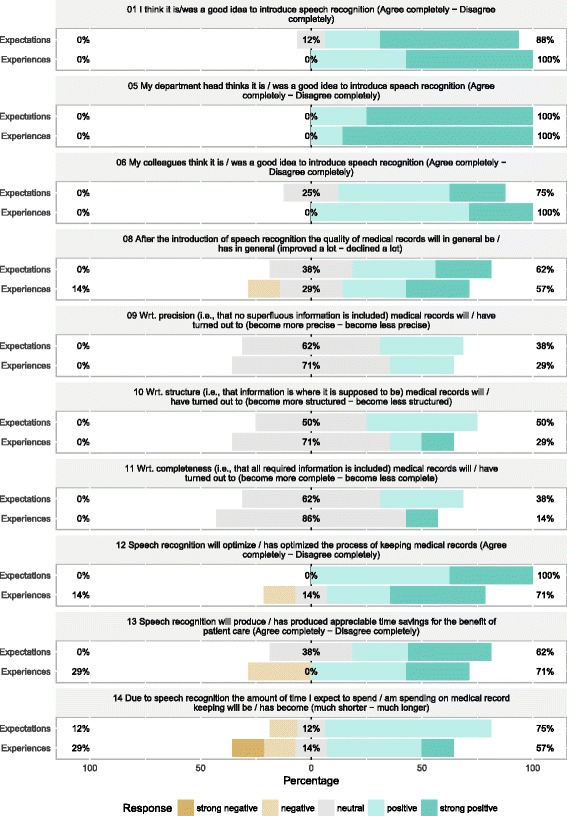
User satisfaction with speech recognition. Results of the survey as proposed by Alapetite et al. [3] as obtained before (*n* = 8) and 10 weeks after introduction of speech recognition software (*n* = 7). Question numbers make reference to the original question numbers. *Green* indicates a favourable outcome; *brown* color indicates a negative outcome. Total percentage for positive (*right*) and negative (*left*) outcome is provided by the lateral numbers. *Grey* indicates a neutral response. Figure produced using likert package in R [20]

Eight physicians provided information at the expectations stage of the questionnaire, whereas seven physicians provided feedback after 10 weeks at the experiences stage (response rate: 87.5 %). The results of the user questionnaire are illustrated in Fig. 2. In summary, it appears that the users in our department were optimistic about the capabilities of speech recognition. This positive attitude was mostly maintained after 10 weeks and the majority of physicians reported appreciable time savings due to the implementation of speech recognition. From the perspective of the physicians it appears as if the process of medical documentation had improved by the introduction of speech recognition (outcomes of Questions 8–12). The majority of physicians did not report on an increased work load after introduction of speech recognition software.

### Turnaround time for the medical document

In total, 2,487 letters were analysed for “time to letter finalised” (in days, provided as median (25^th^ percentile; 75^th^ percentile)). The number of letters analysed ranged from 149 to 223 in the period when documents were produced conventionally from January to June 2015 (sum = 1,156) and from 181 to 291 in the period from July to December 2015 (sum = 1,331) when speech recognition was used.

**Fig. 3:**
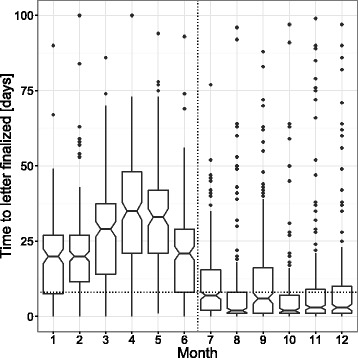
“Time to letter finalised” for year 2015. Vertical dotted line marks implementation of speech recognition. Horizontal line marks median value of the years 2009–2011, when based on internal analysis, documentation process and typist office were running at an optimum. Notches of boxplots provide a 95 % confidence interval for the median. Figure produced using ggplot 2 [21]

In Fig. 3, the absolute change of the median “time to letter finalised” is depicted throughout the course of 2015. In comparison to the values obtained during the first term of 2015 (January – June) when the median time to letter finalised was 26 (14; 35) time to letter finalised was reduced by 85 % in the second term of 2015 (July-December) when time to letter finalised was 4 (1; 12.2). When compared to the benchmarking period, when “time to letter finalised” = 8 (5; 14), the parameter was reduced by 50 % during the second term of 2015. The reduced turnaround time for the final draft, which points towards an increased speed of the whole documentation process, is in line with the observation of others [2]. The raw data to calculate “time to letter finalised” is provided in an additional file [see Additional file 2].

## Discussion

In summary, our results point towards successful implementation of a speech recognition software both concerning user satisfaction and the time spent to create the medical document, suggesting efficient utilisation [10] of the application. It has been argued that change programs fail because not enough focus was put on outcomes [18]. With our approach, these outcomes were already taken into account at a relatively early stage of the change initiative by addressing user expectations and establishing “time to letter finalised” as a performance indicator for documentation speed.

In our opinion, the analysis of the available patient letters and creation of a specific dictionary in order to provide a functional speech recognition software already at the point of software launch was helpful for the successful implementation as it served as a “short-term win” as proposed by Kotter [6]. The size of the dictionary gives an idea of how much training of the software by the individual user would have been necessary before the software would turn out to be fully productive in the case that this dictionary and the shared learning function had not been provided to the users. The analysis of the recent discharge notes of the department underpins the importance of the dictionary as basically all documents created between August 2015 and March 2016 contained dictionary words. Without a dictionary, each user would have had to correct the transcript 9 to10 times (but, with a chance of about 50 % even more, see Fig. 1) until all relevant vocabulary would have been covered. It can be assumed that this would have had a negative impact on user satisfaction. Concerning operational effectiveness, central development of the core dictionary and providing it to all users as well as shared training of the software appear more economical and less time-consuming than having the dictionary created individually during software use by the physicians. It is noteworthy that all necessary adjustments were made using “free” software, which might allow the transfer of our methods to other departments.

The positive results concerning user satisfaction, which are in contrast to observation by Alapetite et al. [3], might, however, be partially due to the advances in speech recognition software during recent years. In the assessment conducted in 2009 by Alapetite et al., the vast majority of users did not perceive that software use was contributing to time savings, which is in line with observations by others [4]. Nevertheless, when regarding the high speech contribution rates that were observed in the “top users” group of Alapetite et al., this is not necessarily linked to technical restrictions concerning possible speech contribution rates at that time. The constellation rather points to a problem with maintaining the motivation to use the speech recognition software continuously, which would lead to improved speech contribution rates and productivity. Remarkably, with our physicians, positive outcomes concerning user experience and satisfaction were obtained even despite an apparently increased work load for physicians concerning documentation tasks. This suggests continuity of the motivation of the software users towards speech recognition. Especially the outcome of questions 13 and 14 highlights that after a period of 10 weeks using the software our physicians perceived this as clearly beneficial for their work, which appears to be in contrast to the analysis of Hodgson and Coiera [4]. When drawing from the framework proposed by Dawson and coworkers [10] other “user/task characteristics”, e.g., the specific workload of the studied physicians or their technical affinity might explain this result. From a process perspective, speech recognition might have provided the physicians with the opportunity to do “their” dictations in a more flexible way, e.g. a functional speech recognition supports the physician to capture the patient’s history right after interviewing the patient.

Our results concerning “time to letter finalised” also point toward an improvement of the whole process by introduction of speech recognition. Noteworthy, “time to letter finalised” is not a measure for the time that is spent for creating a specific document but measures the whole process of creating the first draft. The observed improvements most probably reflect savings of “idle” times when documents where either cued at the typist office or at the physicians desk for the first review.

It is noteworthy that, in contrast to other current developments in speech recognition (e.g., automated transcription of shift handovers), the solution that was introduced aimed at replacing the previously existing typist service at the department by providing the doctor with on-site direct speech recognition, meaning that all documentation was still at least double- checked by physicians at the department and hence the risks for patients due to erroneous transcripts appear to be low.

### Limitations

Our case study does not follow a rigorous scientific approach as presented by Suominen et al. [19], for example, but should rather be understood as a presentation of a methodology with quantitative evidence. A clear weakness of the report is the low number of physicians participating in this study and the lack of a control group. Performance parameters such as speech recognition rate or speech contribution rate during the training period were not made available by Mediainterface, although we initially assumed availability of this data as performance indicators. Our case study was not designed to establish these parameters experimentally. Due to the situation at our department (all physicians are trained with the software now) obtaining this data after the study does not appear to be feasible. However, this point should clearly be addressed in future studies. Due to the uncontrolled design of the study, other confounders explaining the observed reduction in “time to letter finalised” cannot be excluded. Analysis of the amount of documents that were prepared by the physicians highlights that there was no bias arising from systematic variation in this parameter. It can be speculated that the reduction of “time to letter finalised” can solely be attributed to the direct production of the medical documents by the physicians, which saved document waiting times in the typist office. However, even in case of validity of this argument, the questionnaire results (Q13, Q14) where the majority of the users attributed the time savings to speech recognition software support the positive impact of speech recognition. The study, however, cannot draw conclusions of the actual working time spent on documentation.

## Conclusions

With this case study we provide evidence that speech recognition software can be introduced into specialised medical outpatient departments successfully when users are put into focus before implementing the solution. We also provide evidence that contemporary speech recognition software supports the physicians in promptly producing patient discharge letters.

## Additional files

Additional file 1: Commented Script. Commented Linux shell and R syntax of analyses conducted in this article. (PDF 42 kb)
Additional file 2: Raw dataset to calculate “time to letter finalised”. Parameter “time to letter finalised” for each letter that has been sent in 2015 (in days; numeric) and respective month when letter was created (1–12; string). (CSV 16 kb)
