# Supplementary material for: Introduction of digital speech recognition in a specialised outpatient department: a case study
Source: BMC Med Inform Decis Mak. 2016 Oct 18;16:132. doi: 10.1186/s12911-016-0374-4 (PMC5070188; doi:10.1186/s12911-016-0374-4)
Supplement: Additional file 1: — Commented Script. Commented Linux shell and R syntax of analyses conducted in this article. (PDF 42 kb) [file 12911_2016_374_MOESM1_ESM.pdf]

MS-Word documents were converted to easily searchable text files by “libreoffice –headless”. With the command line tool “loconvert” we observed a much lower throughput.

```
libreoffice --headless --convert-to txt:text --outdir /foodir/  foo.doc
```

Text files were split into words, after changing newlines to spaces and capital letters after punctuation marks [:\.!;] to #:

```
cat foo1 | tr '\n\' ' ' >foo2
sed 's/[:\.!;] *[A-Z]/ #/g' <foo2 >foo3
grep -o -E '[#a-zA-Z0-9]+' foo3 | sort > wordlist
```

The resulting wordlist (n = 4,058,099 words) was further analyzed with R:

```
# read the wordlist
# words starting with # are ignored by default
words <- read.table(file="./wordlist", stringsAsFactors=FALSE)

# calculate frequencies of each word
dtw <- as.data.frame(table(words$V1), stringsAsFactors = FALSE)

# ignore less frequent words
# and words starting with digits
dtw <- subset(dtw, Freq > 4)
dtw <- subset(dtw, ! substr(Var1,1,1) %in% c(0:9))

# frequent words (n = 15,883 words)
words <- dtw$Var1

# read SpeaKING dictionary
df_spk <- read.csv(file="FWS-innere.csv", stringsAsFactors=FALSE)
spk <- df_spk$word

# words not known by SpeaKING (n = 2,060 words)
nspk <- setdiff(words, spk)

# write file for aspell
writeLines(nspk, con='./nspk-words')

# check spelling of words
aa <- aspell('./nspk-words',
             encoding = "UTF-8",
             program = "hunspell",
             control = c("-d de_DE"))

# correct words, according to hunspell (n = 787)
knownWords <- setdiff(nspk, aa$Original)

# words not known to hunspell (n = 1,273)
unknownWords <- aa$Original
```

Both wordlists were checked by a physician, resulting in 400 accepted words from “knownWords” 251 from “unknownWords”. After a final comparison against the SpeaKING dictionary (as above) a highly sports medicine specific wordlist of 635 words could be provided to the computing center for addition to our department specific dictionary.
